# Medical and Physiological Problems, Being Chiefly Researches for Correct Principles of Treatment in Disputed Points of Medical Practice

**Published:** 1846-01

**Authors:** 


### Art. VI.

*Medical and Physiological Problems, being chiefly Researches for Correct Principles of Treatment in Disputed Points of Medical Practice.* By William Griffin, M.D., Member of the Royal College of Surgeons in Edinburgh, one of the Physicians to the County of Limerick Infirmary, &c.; and by Daniel Griffin, M.D., Member of the Royal College of Surgeons in London, Assistant Physician to the County of Limerick Infirmary, &c. *London,* 1845. 8vo, pp. 356.

The following remark appears in the preface to this publication:

"There are two modes of treating all diseases, a right and a wrong one; and it is popularly believed that the science of medicine has long since determined between them; that in every dangerous case, or, at all events, in those of ordinary or frequent occurrence, the practitioner has only to refer to received principles or authorities on the subject, and that if he commits an error in his selection of remedies, it is entirely attributable to his want of information or ability. The uninitiated are little aware of the deep responsibility that rests with the young practitioner, when a decision is demanded of him on such occasions, or the profound judgment, as well as extensive information required to arrive at a correct conclusion between conflicting authorities of equal consideration."

It occurred to one of our authors [we cannot say whether it was the Castor or the Pollux of the Irish profession, as no signature is attached to the preface,] some years since, that it would be a most useful, practical, and interesting study to collect cases with reference to all the most important of disputed points in practice, and afterwards, as a sufficient degree of personal experience happened to be attained on any one of them, to review the opinions of all the best authors on the subject, and endeavour to solve that most difficult question—what is the correct principle of treatment? The idea was excellent, and the results of its accomplishment are in the work under consideration.

The first four problems are so closely related to each other, that we shall take them together. They virtually refer, as might be expected from the previous publications of the authors, to the diagnosis and treatment of spinal irritation, although the first seems to have no connexion with that form of disease. The four problems are:

"1. What principles should be kept in view by the physician in the treatment of enteritis?"

"2. How are nervous affections distinguishable from inflammatory?"

"3. What is the diagnosis of abdominal inflammations?"

"4. In spinal irritation, is there really any affection of the spinal cord, or of its membranes?"

Enteritis.—*Treatment.* The discussion of the first problem is not to the point. Its object is to show, that enteritis is best treated by opium. The history of a case so handled is preceded by extracts from systematic writers as to treatment by bleeding and purgatives, tending to show considerable discrepancy of opinion as to *extent* at least of depletion, and as to the time and mode in which purgatives should be administered.

Parr and Good advise moderate depletion as less likely to be followed by gangrene; Abercrombie advises more vigorous measures, and Elliotson directs the patient to be set upright, and bled "from a large orifice without mercy." The tone of confidence and decision usually adopted by the latter writer has gained him many disciples, but we suspect that mature experience will very much shake their trust in their leader, and we would refer to the last dictum of bleeding "without mercy," as one likely to be questioned. It is well known that cases of enteritis may be cured safely and quickly without any depletion whatever.

Authorities take two sides on the use of purgatives in enteritis. Parr, Pemberton, and Good recommend mild purgatives at first; then, if necessary, the more active. Dr. Elliotson is confident, *more suo,* and we think most dangerous in his advice. "We should first," he tells us, "bleed freely, because purgatives will not operate until we have done that; we should then give a large dose of calomel, such as a scruple, by the mouth, and a strong purgative injection, with plenty of salts, or salts and senna, or colocynth, or oil of turpentine, and repeat the calomel in ten grain doses every four or six hours, *giving purgatives in addition from time to*

*time.*" When we consider how readily enteritis may be mistaken for ileus, or spasmodic cholic, the danger of such treatment is manifest. Dr. Abercrombie recommends that the purgatives be not administered in the early stage. This plan of treatment is that, we believe, which the majority of modern physicians adopt.

We will now give an abstract of the case before referred to.

A lady, aged 32, affected with symptoms of enteritis, was bled to eight ounces, and two dozen leeches applied to the right hypochondriac and iliac regions. Two grains of calomel and one of opium were given every second hour. No relief following, the next day twenty ounces of blood were taken from the arm; aloes and henbane given to operate on the bowels, and a terebinthinate enema administered.

"On administering this last, the patient was seized with a dreadful forcing or bearing-down pain in the rectum, and passed nothing; the pain seemed as excruciating as any that could occur in violent labour, lasting for about twenty minutes, and was then relieved by the warm bath. In two hours afterwards, the administration of a simple injection of oatmeal tea was followed by similar suffering, and was in like manner retained. The permanent pain was at this period severest in the left iliac region and about the navel, where the tenderness on pressure was extreme; the countenance was more anxious; the tumidity of the abdomen was increasing, and the stomach beginning to reject the drink. In consultation with another physician, it was now agreed to take blood again, and eighteen ounces more were drawn, being the third general bleeding within twenty-four hours. Two grains of opium and a grain of calomel were given immediately after, and ordered to be repeated every two hours through the night. In the morning (the 4th) there was a considerable improvement; the abdominal tenderness was diminished, the pain and sickness of the stomach had very much subsided, and the injections had come away with some dark, thin, feculent matter. She still, however, felt pain and a sense of great weariness at the lower end of the sacrum, shooting up through her back, and she had a great difficulty in passing water. She now informed me that a few days previous to her present illness, she was attacked with a profuse leucorrhœal discharge, attended by heat and sense of scalding, but that it had since abated or almost ceased. A fomentation to the lower part of the abdomen was ordered, and the opium was continued in two-grain doses every two hours, without calomel. In the evening the improvement appeared progressive; the skin was cool, the pulse soft at 110, the tongue cleaner; the abdomen was still full, but it had nearly lost its tenderness, and she could turn in the bed with little pain" (p. 5.)

It was now discovered that there was a purulent discharge from the rectum:

"On the next evening, as she lay on the sofa while her bed was making, she felt a solid substance passing from the rectum, which alarmed her terribly. It was found to be a rope of sloughy stuff, soft and purulent outside, but tough and fibrous within, not unlike the ischiatic nerve in a decayed state, suspended from the rectum for the length of a foot or more. On attempting to draw it away, it appeared to be still adherent within the gut, and she complained of pain. After a little, however, it was removed without much effort, and a gush of matter to the amount of perhaps two tablespoonfuls followed. The slough was about the thickness of the thumb or more, and was fifteen or sixteen inches in length. We at first supposed it was a portion of the small intestine which had mortified and been thrown off; but on close inspection no distinct traces of a canal could be found. Some time after an injection of warm water and sweet oil was administered, which came away in about twenty minutes mixed with some matter, but without any appearance of fæces. On examining the rectum, a rugged irregular edge was felt on the posterior side, close to the sacrum, as if it was the termination of the part from which the slough had been cast off; the examination gave much pain, especially when the intestine was pressed upon within. Several days passed without much alteration in the case: there was matter daily discharging to the amount of three or four ounces, and there was at times severe dysury, at last demanding the use of the catheter." (p. 7.)

Three days having elapsed since the pain and tenderness of the abdomen was experienced, and six since the bowels were moved, an aperient was requisite, and a dose of castor oil given, followed by pills of aloes and henbane. All the symptoms now recurred in a more aggravated form. There was increasing distension of the abdomen, the pulse became feeble and rapid, the thirst extreme, the vomiting frequent, the countenance was sunken, the look anxious, and the face covered with a clammy perspiration. Three grains of opium were given as a first dose, and two every second hour afterwards; a dozen leeches applied to the abdomen, and fomentations with decoction of poppy-heads. The effect was wonderful; the pain and tenderness gradually abated, the pulse became slower, the sickness ceased, and the expression of countenance improved. Matters went on well until the seventh day from that on which the bowels were last moved, without any evacuation. At that period, however, (as Dr. Griffin anticipated,) "the tenderness in the left ilium was again felt, and it was soon followed by pain and feverishness, with a disposition to vomit."

"There was now no doubt on our minds that the recurrence of the attack was attributable to distension and not to perforation of the intestine, as we had apprehended. After giving a large opiate, therefore, she was ordered a few grains of calomel, with mild doses of castor oil and jalap every second hour, until the bowels were freely moved. Great relief was obtained, but the pain and tenderness of the abdomen finally subsided on resuming the opiates for twelve or fourteen hours after the purgative had ceased operating."

The case now gradually but slowly advanced to recovery:

"A mild purgative was given at the farthest on every fourth day, which operated without creating pain or uneasiness, and by diminishing the interval gradually, the bowels were after a little brought to act daily with a small dose of rhubarb and cascarilla. She was still, however, unable to sit up in the bed, or to turn to either side on account of the excessive soreness inside the sacrum. The motions continued to be smeared with matter; sometimes small bits of fresh slough came away; sometimes spoonfuls of healthy pus with stuff like jelly. Weak sulphate of zinc injections, and even those of simple water were made use of, but they gave great uneasiness, and served to do more harm than good. At this time, about eight weeks from the commencement of the attack, she became very hysterical, got fits of crying and laughter, which lasted for hours, and was sometimes slightly delirious. She had been kept very low all through her illness, but was now allowed nourishing diet, meat, and a little wine; there was an immediate improvement in all the symptoms; her strength and health mended; her mind became cheerful; the discharge of matter diminished, and at last was only occasionally observable. The soreness about the sacrum was also lessened so considerably that she was able to dress, lie on the sofa, and sometimes sit up for a short time. At the end of three months she could move about the room a little, and at the termination of the fourth she was perfectly recovered." (pp. 10-11.)

In commenting on this interesting case, we may fairly award to Dr. W.

Griffin the merit of saving his patient's life; but we should have been glad to have learnt more about this sloughing of the rectum. Why did that excruciating, dreadfully forcing pain come on immediately after the administration of the terebinthinate enema? *Is it possible that the nurse perforated the bowel with the enema-tube?* How was it that distension of the bowel in the left iliac region caused the sloughing of the rectum, and the rugged, irregular edge at the posterior side close to the *sacrum?*

With regard to the principles of treatment in enteritis, Dr. W. Griffin is in favour of a free and early bleeding, and if this fail, bleeding must be abandoned as the main resource, and employed as auxiliary only. With regard to purgatives, he observes that in the greater number of cases of enteritis, the favorable termination is by a free evacuation of the bowels, and that before this occurs relief is seldom obtained. To the experience of this strong fact may be referred, he thinks, the popularity of the purgative treatment. But Dr. W. Griffin shrewdly remarks:

"Effects are too often, in the science of medicine, mistaken for causes. When cholera first appeared in this city calomel was profusely employed in its cure, and it was eventually found that patients who became salivated almost invariably recovered. This was esteemed proof positive of the efficacy of the treatment, and mercurials became more popular than ever. I found, however, on examining the registries of the hospital with which I was connected, at the termination of a month, that in the stage of collapse no more than one patient in ten could be brought under the influence of mercury, so that there were only four recoveries in forty. This told little for the remedy as far as cases in relapse were concerned, and I immediately set about ascertaining what the amount of spontaneous recoveries might be in the same stage. From all I could gather from the experience of others or my own, I began to suspect that they would reach nearly the same amount; and at last I became perfectly convinced that the actual fact was, *the patients did not recover because they were salivated, but they were salivated because they recovered.* Mercury in any shape, in the stage of collapse, was thenceforward discarded from my practice in the hospital, and though it excited some observation at the time, the subsequent experience of the profession at large bore me out in the decision. I cannot but feel that somewhat of the same error prevails with respect to purgatives in enteritis; the disease is not a very common one, and the experience of an individual could scarcely warrant him in offering opinions at all confidently, when they are opposed to general practice; but certainly all the information I can glean, or the experience which has fallen to my share, would dispose me to say that, in intestinal inflammation, the relief obtained is seldom the direct effect of the purgative, and that *people do not recover because they are purged, but they are purged because they recover.*" (pp. 15-16.)

After a perusal of the preceding case our readers will readily imagine that Dr. W. Griffin is a warm advocate for the treatment of enteritis by full and frequently repeated doses of opium, and details several cases at length as illustrative of its success. There is some doubt on our minds as to the diagnosis of many, if not most of the cases of enteritis, and this doubt introduces us to the two next problems: How are nervous affections distinguished from inflammatory, and what is the diagnosis of abdominal inflammations?

*Diagnosis.* The term enteritis is, we believe, now generally understood to mean inflammation of that part of the peritoneum constituting the outer coat of the intestines. The characteristic symptoms of this as well as of other forms of peritonitis, are pain and tenderness on pressure of the abdomen. Dr. W. Griffin urges that these symptoms are really fallacious in diagnosis; that they exist in an exquisite degree in affections of the spinal cord or its membranes; and that the diagnostic sign of the latter class is tenderness on pressure at the portion of the spinal column corresponding with the disturbed organ. The following paragraph expresses so clearly Dr. W. Griffin's opinions, that we subjoin it at length :

"There are three effects very common to inflammation, or disorder in the spinal cord, or at the trunks of its nerves. First, superficial tenderness, more or less exquisite, and either limited to the integument immediately over or about the affected portion of the cord, or extending thence to the front of the abdomen or thorax, in the direction of the spinal nerves which have their origin there, or in the ganglionic nerves supplying the viscera, which have connexion with that portion of the cord. Secondly, loss of power evinced in partial or complete palsy of the parts or organs to which the affected nerves are distributed. Their effects often occur simultaneously, but any one of them may also occur independently of the existence of the others, offering very strong evidence that the sensibility of the surface or skin, and that which exists in internal organs, is dependent upon nerves, which though sentient, are as distinct from one another as they are from those on which the power of motion depends. Keeping these ordinary effects of disorder of the spinal cord in view, it must appear obvious, when we detect soreness or tenderness on pressure in the region of the liver or spleen, or in the lower abdominal viscera, how important and essential it is for us to ascertain whether the soreness be superficial or deep-seated, which we can always do by careful examination. Again, when pain is complained of in the region of the liver, or middle or lower parts of the abdomen, how necessary it is to ascertain whether, as in the case of soreness, it be superficial, and if deep-seated whether it be merely an affection of the nerves of the part, and probably connected with some affection of the adjoining portion of the spinal cord; or whether the internal organ itself be in a state of acute or chronic inflammation. Finally, if there be oppression in breathing, whether it arises from deficient action in the respiratory muscles, or the imperfect performance of the process of oxygenation of the blood in the lungs, or from actual inflammation, or organic disease of the mucous membrane, or parenchyma of these organs. Or if there be obstinate constipation of bowels, whether it depends on spasm or enteric inflammation; or whether purely on deficient power, or partial palsy of the muscular fibres of the intestines. I know of no case which so often deceives inexperienced or uninformed practitioners as obstinate constipation of bowels from paralysis of the nerves, when connected, as it frequently is, with abdominal pain and exquisite soreness on pressure." (pp. 49-50.)

It seems to be generally allowed that the first symptoms of ileus or colic resemble those of enteritis. There is usually the same tenderness on pressure, the same obstinate constipation, the same kind of twisting pain with vomiting, and anxious expression of countenance. These symptoms are consequent on all violent stimulation of the intestinal canal. It is only, in fact, by the *termination* of the attack that ileus differs from colic and enteritis. If the spasmodic action constituting the disease relaxes speedily, and the transit of the fæces along the canal is re-established, then it is only a case of colic; but if the spasm becomes persistent, the compacted fæces, already acting as an irritant, continue that irritation, and ultimately inflammation of the intestine, extending to its peritoneal coat, is set up. It is manifest, then, that in such cases opium and its adjuvants must be the key-stone of the plan of treatment; purgatives will only add to the irritation. The leading indication is to unlock the stricture of the bowel, either by opium or copious mild enemata. Unless this be done, enteritis will as assuredly follow the retention of the fæces, as cystitis follows retention of the urine in spasmodic stricture of the urethra.

In what morbid condition will this spasmodic stricture of the intestinal canal originate? It may be and probably is always connected with irritation of the muscular fibres of the canal, through the medium of the nerves distributed on the mucous surface. This irritation may depend, firstly, on the nature of the irritant. An alvine concretion, an indurated mass of fæces, or a large accumulation of the latter in the colon, as in the case before detailed, will induce a sufficient amount of irritation and consequent spasmodic action. We think the last mentioned is the most frequent irritant of all, as in most of the cases that we have witnessed relief has followed on repeated and enormous evacuations. Secondly, the exciting cause of the irritation may be in itself trifling, but there may be a too great susceptibility of (in Hallian phrase) the incident-excitor nerves of the muscular structures of the intestines, and thus spasm of those structures be excited, just as tetanus or trismus arises from impressions on the skin. Now the question arises: 1st, whether this susceptibility is connected with "irritation" of the *spinal* ganglia, or of the *sympathetic* ganglia; and, this being ascertained, 2dly, whether that susceptibility is *invariably* or even usually conjoined with a similar irritability of the spinal sensitive nerves, or the sensory tract of the spinal cord? In tetanus, we know that the sensitive nerves of the surface are not more than usually susceptible; impressions upon them excite spasm, but no pain. The seat of the irritability is in the motor tract; the pain is in the affected muscles. By parity of reasoning we may infer that the pain of colic is seated in the muscular structures of the bowel, and not in the nerves of the mucous membrane or of the skin.

According to these views, neither irritation of the spinal cord nor of the sympathetic ganglia, is *necessarily* accompanied with increased sensibility of the skin or mucous membrane of the intestinal canal, and consequently that spinal tenderness is not diagnostic of spinal irritation, nor of those spasmodic affections which originate in some morbid condition of the spinal cord. It can only be considered so when the morbid change is not limited to the motor tract, but extends as well to the *sensory*.

That the muscular contractions of the intestines depend in a great degree upon the sympathetic ganglia and their twigs, is manifest from the persistence of the peristaltic motions after entire removal from the body; consequently, it is not clear to us that the spasmodic affections of the viscera, supplied with sympathetic nerves, are in all cases connected with irritation of even the motor tract of the cord. The *ganglia* may be the primary seat of the irritation, and the *sensory* tract irritated secondarily.

We need go no farther than Dr. Griffin's Essay for pathological proofs of our views. We subjoin two cases, the one of enteritis, according to Dr. Griffin's diagnosis; the other a case of spinal irritation:

I.—*Case of enteritis.* "A strong active man, aged 30 years, was seized with pains in the *abdomen, chiefly about the situation of the umbilicus; the pain came on more violently at short intervals, but never ceased,* and was attended with *extreme soreness of the abdomen,* especially in the situation of the pain. The stomach was sick. He got castor oil and turpentine, which was vomited up again. He afterwards got repeated doses of calomel and colocynth with injections, but the former produced no effect, and the latter came away as they were administered, without any admixture of fæces He was subsequently blooded to the amount of thirty ounces or upwards, with some little relief to the pain, and the purgatives were continued, but after some short time all the symptoms became worse. About ten o'clock at night the patient's friends became exceedingly alarmed at his increasing illness, and desired a consultation; upon which occasion I was requested to see him.

"I found him lying on his back, *moaning faintly*, and complaining of constant pain about the umbilicus, which at intervals of a few minutes increased to a violent degree,—*he had frequent retching, and could retain nothing solid or fluid upon the stomach;* his countenance was dejected; his pulse 100; his skin rather warmer than natural. He had had no movement of the bowels since the commencement of the attack. The centre of the abdomen was covered with a blister, so that I could make no examination as to the degree of tenderness; but all the parts above, below, and at the sides beyond the margin of the plaster, were excessively sore to the touch. The medical gentleman in attendance, finding all his efforts to get the bowels moved were unavailing, was just preparing to give him some croton oil.

"I represented to him how very unlikely it was that by any purgatives he could get an inflamed bowel to act; that inflamed muscles never do. That the chief object appeared to me to be to subdue the pain and inflammation, leaving the evacuation of the bowels entirely to nature, which would probably effect all that was desirable when not interfered with by an inflammatory condition of the parts. I proposed that the blister, which had been on only about two hours, should be removed, and that eighteen or twenty leeches should be applied about the umbilicus, where the pain was most acute; that warm fomentations should be afterwards applied both to allay the suffering and encourage bleeding from the leech-bites, and that *two grains of opium with two of calomel should be given immediately, with one grain of each every hour afterwards*, until the patient fell asleep. On the medical attendant expressing some alarm at the quantity of opium which might be given in this way, and the bowels obstinately constipated, I observed, that if sleep could be procured, the man would probably awaken freed from all pain, and have his bowels moved without the necessity of a purgative.

"The leeches were applied, and the bites bled freely with the fomentations. *The opium remained on the stomach, and soon after the patient took the third dose the pain evidently abated.* In less than an hour he became easy, had a heavy look, and before the hour came round for the next dose was in a sound sleep. *At seven o'clock in the morning, after six or seven hours uninterrupted sleep, he awoke freed from pain or sickness*, and on being assisted to the night-chair, had a free, easy, and copious evacuation. He had one or two more motions in the course of the morning, and required no further treatment." (pp. 30-31.)

II.—*Case of spinal irritation.* "A young woman, aged 25 years, was attacked with pain in the bowels at night, after a feeling of chilliness. She took some essence of peppermint and went to bed, but the pain gradually increased, and at two o'clock in the morning she took twenty drops of laudanum. At seven o'clock, the pain still continuing, she took castor oil, with ten or fifteen more drops of laudanum, by the directions of an apothecary, and soon after ten drops were repeated in a saline draught I saw her at one o'clock, and found her *writhing with pain chiefly round the umbilicus, and to the right side. It became more violent by fits, like colic, though never entirely subsiding;* and during the intervals of comparative relief, she sometimes threw her arms about restlessly, sometimes lay as if insensible, with the eyes turned up, and the lids half open. *Her complaints were low, scarcely audible;* her respiration painful when deep. Turning from side to side increased her pain, although when the paroxysm occurred she turned sometimes on her face. *There was excessive tenderness of the*

*abdomen*, the least pressure making her scream. *She had been constantly vomiting for the last few hours.* The castor oil had operated once scantily; her pulse was but little quickened, and there was no heat of skin.

"Here was a case of constant pain in the abdomen chiefly about the umbilical region, liable to severe exacerbations, attended by exquisite tenderness on pressure, vomiting and constipation, and continuing for twenty-four hours. I am convinced that almost any young physician would have felt great difficulty, indeed almost an impossibility of determining, from a consideration of the symptoms, that the complaint *was not inflammatory;* and I believe that the great majority of either young or inexperienced ones would at once infer the existence of inflammation, and bleed. I say so from having witnessed it, and from having early in my own professional life always prescribed in such cases with timidity, as if I felt that all consideration of the symptoms led to little better than conjecture. I had now, however, new means of diagnosis in the state of the spinal column, on examining which my mind was set at rest. As soon as I pressed on the upper lumbar vertebræ the girl started violently, caught my hand, and complained that I had hurt her dreadfully; the pressure, she said, had increased the abdominal pain. She had never had any hysterical attack in her life. I ordered the abdomen to be fomented freely, and gave her *five grains of calomel with half a grain of opium*, directing five grains of aloes and five of extract of henbane to be taken every two hours. The calomel and the first dose of the aloetic pills remained on the stomach, but the succeeding doses were rejected; a purgative draught was also thrown off. She then got a purgative enema, which was repeated in an hour, but both passed off without any appearance of fæces. The vomiting, pain and tenderness of the abdomen, continued very severely throughout the evening. At night she got a turpentine enema, and was placed in a hot bath, which gave some relief and procured a scanty evacuation. The pain, however, soon recurred, and *she was ordered a grain of opium, to be repeated every hour until it should subside.* The bowels were freely moved soon after taking the first dose, but *she did not experience any considerable relief until she had taken the third, after which she fell into a sound sleep, and in the morning was in every respect improved.* The pain had altogether subsided, and the exquisite tenderness was now felt in the right epigastric region only. She had threatenings of a return of the attack in the course of the day, but it was readily subdued by a repetition of the opiate. On the following morning there was scarcely any pain or tenderness; and if the complaint had been inflammatory, I would now have left the patient's bowels perfectly at rest; but believing it to be an affection of the spinal cord, arising from disorder of the digestive functions, I ordered another dose of castor oil, which operated freely, and was followed by no recurrence of the attack." (pp. 40-41.)

Upon a comparison of these two cases, it will be seen that in their symptoms, course, and termination, they are identical. To avoid repetition, we have underlined passages in each which are almost parallel. It is true, in the one case there was no spinal tenderness, and in this no leeches were applied, or calomel administered in repeated doses, as in the other case; but in the accompanying examples of spinal irritation detailed by Dr. Griffin, simulating enteritis, we find both these means were conjoined with the opium. The principal circumstances to be observed are these: firstly, that opium is given equally in both classes of affections, and with equally beneficial results; secondly, in the cases of enteritis, that is, in those in which there is no spinal tenderness, a general bleeding is practised. These latter cases may indeed, after all, have been cases of spinal irritation," as Dr. Griffin acknowledges that spinal tenderness is sometimes absent. We think Dr. Griffin might safely treat cases of this kind in the same way as he treats cases of the former; that is to say, omit the general bleeding. Terebinthinate and all other stimulating enemata should also be avoided as injurious, and copious lavements of simple warm water, or medicated with æther or laudanum, should be substituted. These, conjoined with full doses of opium, leeches, and fomentations, we have invariably found to be successful in cases like those discussed by Dr. Griffin. The spasmodic stricture will sooner or later relax, the pain will cease, and copious evacuations take place. We shall recur to the consideration how far opium may be safely administered in diseases of the spinal cord, in the discussion of the next problem.

Problem IV. *In those disorders which have been termed spinal irritation, is there really any affection of the spinal cord or its membranes?* The solution of this problem comprises a criticism of a critic. We gather from this chapter that Dr. Griffin, to use his own words, really knows nothing at all of the nature of spinal irritation, or of the pathological condition with which it is connected. There are no opportunities of investigating its pathological anatomy, and if there were, it is doubtful, Dr. Griffin thinks, whether any alteration of structure could be specifically connected with the symptoms; instancing the several forms of mania, epilepsy, and chorea, as analogous. There is certainly, however, "a morbid condition" of that part of the spinal cord corresponding to the seat of pain; and in proof of this proposition, Dr. Griffin gives a tabular summary of 148 cases in which there was tenderness of the cervical, cervico-dorsal, dorsal, dorso-lumbar, and lumbar regions respectively, with simultaneous functional disorder of the viscera corresponding to these regions. The solution of the problem is however imperfect, for we have no information afforded us as to the origin or nature of this morbid condition. Dr. Griffin has, in fact, failed to illustrate the class of affections to which he has devoted so much attention, by the more accurate spinal anatomy and physiology of the day. This defect is the more inexcusable, as since he first wrote on the subject one or two complete monographs have been published. For a short synopsis of our own views, we would refer to p. 96 of our 37th number; and for some views regarding the anatomy and physiology of the spinal cord, (and without a thorough knowledge of these it is impossible to understand the pathology of spinal irritation,) we would refer to our 34th number. The causal diagnosis is all-important in these cases, with reference to the treatment. Thus the neuræmia, arising from the incident excitor action of the ovaria on the spinal cord, may not be confined to the lumbar region, but, as Dr. Laycock has clearly shown, may affect the dorsal and cervical region, or even certain portions of the brain, in consequence of the extensive affinities of the ovaria to the nervous centres. Now, in such examples (as hysterical cunning, pica, &c.) a merely local treatment cannot be consistently recommended.

To furnish another example: gout in females assumes almost always the anomalous form; it most rarely attacks the serous or sero-synovial structures of the toe; more frequently those of the joints of the fingers; probably quite as frequently those of the spinal column. We should therefore be on our guard against cases of this kind, by a close investigation of the constitutional peculiarities of the patient, and we shall thereby escape treating arthritic or rheumatic inflammation of the spinal meninges, or articulations, as if simply cases of neuræmia, induced by an incident excitor action of the kidneys, ovaria, or other viscera on the spinal ganglia. The same method will assist us in distinguishing myelitis as well from spinal irritation as from visceral inflammation. Dr. W. Griffin relates an instance of myelitis, the leading symptoms of which closely resembled those of enteritis. The captain of a merchant-vessel, a hale, hardy man, was attacked by pain in the back and belly, with obstinate constipation. After undergoing bleeding and purgation, Dr. W. Griffin was called into consultation, and found the patient complaining of great pain in the abdomen and round the loins, with difficulty of passing water, and obstinate constipation. The entire surface of the abdomen was very tender to the touch, and in the pubic region excessively so; there was also over nearly the whole trunk of the body an acute soreness of the skin, especially about the hips and lower extremities, which made it painful to turn himself in the bed, or to permit any one to assist him in doing so. There was great tenderness on pressure along the whole spinal column, but he complained of the pain only in the lumbar portion, and in the corresponding parts of the abdomen in front; his skin was hot, his tongue foul; his pulse, small and rather feeble, at 128. He had been bled to the amount of more than a quart the night before, and was continuing the purgatives and enemata. The treatment Dr. W. Griffin recommended was such that the patient took twelve pills within the same number of hours, containing two grains of calomel and one grain and a half of opium in each, without relief to the pain. Pills of crude opium (one grain each) were then substituted and continued for twenty-four hours, and at last acetate of morphia and mixture of henbane. Sleep now came on, and the pain abated. At the next visit, however, symptoms of softening of the cord made their appearance; the left arm was paralytic and tender to the touch; in five or six days after the right arm was paralyzed. Subsequently, each of the lower extremities became successively affected.

Dr. W. Griffin was of opinion from the first that the disease in this case "was some inflammatory affection of the spinal cord or its membranes." Acting upon this opinion he prescribed the calomel and opium, as already stated; but as a placebo for his colleague, "two dozen leeches were applied to the pubic region instead of the lumbar vertebræ," to which, in Dr. Griffin's opinion (and we may add in ours), they ought to have been applied; and shortly afterwards "consented to the anxious desire" of his colleague to apply a blister to the abdomen. It is manifest that the treatment of this case was wrong. We need say nothing as to the use made of the leeches and blister. No placebo should be administered to a colleague at the expense of the blood and ease of the patient. As to the opium, Dr. W. Griffin shall be his own judge. The following is an extract from the discussion of the problem, *What are the therapeutic effects of opium?*

"There are some inflammatory affections to which, after all, it does not appear that opium can be ever advantageously applied. One of its direct and obvious effects given in large doses to healthy persons is to produce congestion of the brain, and *probably in the spinal cord.* Of this we have sufficient evidence in the apoplectic symptoms it occasions, in the relief of these symptoms by large depletion; and again in the recovery of persons dying of hæmorrhage by large doses of it, which I conceive is effected purely by the slight congestion it is then capable of inducing, restoring the healthy tension of the vessels in the brain, and so preventing syncope and death. When given in acute inflammatory affections of the brain, therefore, its tendency is to produce a state of circulation in the organ, which must rather aggravate than diminish the mischief going on there. I believe *this is true also in inflammatory affections of the spinal cord,* since it is only on that presumption I can at all account for its total failure as a remedial agent, in some cases which have fallen under my observation, and in which it got a fair and full trial." (p. 137.)

Problem V. The next problem is propounded and discussed by Dr. D. Griffin: "*Under what circumstances, and to what extent, is bleeding proper in diseases of the brain?*" By the term, diseases of the brain, Dr. D. Griffin more especially means apoplexy and apoplectic affections, an indefiniteness of expression sufficient to raise an expectation of indefiniteness in the writer's views. And it is even so. Dr. D. Griffin first shows the contradictory opinions of various writers as to the utility of bleeding in cases of apoplexy; and he attributes this circumstance to varieties of structural changes now known to take place in the brain, and formerly to a great extent unknown. Dr. D. Griffin mentions "one conclusion of immense practical importance," namely, "that in considering the expediency of bleeding, *the actual degree of organic disease that exists* is of infinitely more importance than its seat or nature,—in other words, that bleeding is badly borne in all cases of extensive organic disease of the brain, *wherever the disease may be situated or whatever be its nature.*" Now, on the opposite page, Dr. D. Griffin observes, that "symptoms cannot be depended upon as indicating the particular place, or the *actual amount* of cerebral disease;" we therefore cannot see how this conclusion can be of such immense practical importance.

Dr. D. Griffin is of opinion that large bleedings are improper in all affections of the brain attended with severe and protracted pain, as such cases usually die from exhaustion induced by their sufferings.

Not one word is said of functional disease of the brain, so much more common than structural diseases; as for example, those consequent on disease of the kidneys and heart.

Problem VI. "*On what morbid states does the occurrence of coma and sudden death in jaundice depend?*" Dr. W. Griffin discusses this problem and comes to this common-place conclusion: "If, with such imperfect materials, even a conjecture might be hazarded, I should, on the whole, be disposed to say, that the cerebral affection is rarely the primary disease, but is superinduced, we know not how, by the suppression of a most important excretion, as it sometimes is in the suppression of the catamenia, and almost always of the urine." This problem is discussed without any reference to the important fact, that in many cases of jaundice a large proportion of the bile carried into the circulation is excreted through the kidneys.

Problem VII. "*Is the law of visible direction, as at present received, a true one?*" Dr. D. Griffin deduces, from various ingenious experiments, a solution in the negative. He thinks, "we must, for the present, rest satisfied with the simple statement, that when rays of light fall on any point of the retina, that point has the property of representing the object from which they come in *its true direction,* without any regard to the obliquity of their incidence."

PROBLEM VIII. "*Is laryngismus stridulus, or the crowing disease, a spasmodic or paralytic affection?*" This essay is a detailed criticism on the late Dr. Hugh Ley's views as to the pathology of that disease. The objections are well put, although we believe the refutation of Dr. Ley's theory is in the present day unnecessary, as few practitioners found their plan of treatment upon it. Dr. W. Griffin shall speak for himself as to the solution of the problem.

"The foregoing observations must sufficiently display the error Dr. Ley has fallen into with regard to the effects of palsy of the recurrent nerves in occasioning the phenomena of laryngismus stridulus. On the whole, after all the consideration I have devoted to the complaint, and having, I think, given other ingenious arguments of Dr. Ley their full weight, I must still confess myself a disciple of the older doctrine, that the affection is one of spasm or partial convulsion, like cramp, rather than of paralysis. The fact of its being frequently benefited by antispasmodics, with which Dr. Underwood tells us he latterly cured most cases, and by anodynes, as opium, hemlock, cicuta, &c., recommended by all modern writers on the disease, favours this view; the circumstance of the sudden occurrence of the gasping and crowing on washing with cold water, laughing, crying, or agitation of mind, also supports it, as well as the almost universal coexistence of the carpo-pedal contractions, and the frequent termination of the complaint in convulsions. But above all these, as a strong analogical evidence for its spasmodic character, I place its paroxysmal nature, and the manner in which the paroxysms occur. The office of the superior laryngeal nerves would lead us to expect a disposition to spasmodic action on the least irritation or excitement, but far more on the increase or decrease of the susceptibility of the parts, and disposition to spasmodic action. . . . . One can well understand how dangerous any morbid increase of the sensibility of such nerves at their extremities, or any existence of irritation at their origin, might prove; why the danger should occur in irregular paroxysms, and why the exciting cause which occasioned them on one day should be altogether powerless on the next. If it be inquired further, why such a dangerous result as the suspension of respiration in the crowing disease does not occur more frequently, it can only be replied, that we are wholly ignorant of the morbid condition which disorders the functions of those nerves, or whether it exists at their extremities, or their origin in the medulla oblongata. If we suppose the affection to be organic, we should find it more difficult to account for the occasional recoveries under very mild treatment, than the usual fatality under the most active. If it be functional, and therefore symptomatic, we can better understand why it might depend on a variety of causes; at one time upon an affection of the head, at another of the bowels, at another upon dentition; we can comprehend, too, how these several affections, influencing peculiar predispositions, may in one child occasion hydrocephalus, in another convulsions, in a fourth that more rare infantile disorder, the crowing disease." (pp. 135-6.)

We are rather surprised that Dr. W. Griffin has not seen the analogy between this affection and cases of spinal irritation. A more thorough acquaintance with the physiology of the spinal cord would have enabled him to understand, how the changes induced on the jaws during the process of dentition would exercise an incident excitor influence on the central axis, and develope there the neuræmic state in those predisposed. He would also have understood the true bearing and significancy of statements like the following: "why are not grown children attacked with laryngismus stridulus? I have never seen the complaint except in mere infants; all Mr. Robertson's and most of Dr. Ley's cases appear to have been under two years of age." (p. 131.) The process of dentition is the external index of a general change in the system,—it is a stage of development; and it is for this reason that laryngismus and other spasmodic affections are more frequent during dentition, as attacks of spinal irritation most usually occur in females about puberty. As the predisposition is the greater, so is the attack the earlier; and in those highly predisposed, it will often appear with the *premonitory* symptoms of dentition. On the other hand, it not unfrequently occurs during the *third* year, when the molares are protruding.

PROBLEM IX. "*Does suffering necessarily imply self-consciousness? Are sentient beings necessarily percipient?*" Dr. W. Griffin draws a distinction between mental consciousness and sensation. The former "implies, not only the perception of thoughts and sensations, but the reference of these to something that remembers the experience of the former thought or sensation, which believes it existed before the present moment, and that it was itself which experienced all." Sensation consists in a mere sense of the present, neither including remembrance of the past nor anticipations of the future. This may be divisible; the other is indivisible. It is thus that Dr. W. Griffin, in rejecting the reflex theory, explains the facts on which that theory is based. A decapitated tortoise, when irritated, conceals itself beneath its shell: this it does because it is a *sentient* being; it has no will, no memory, no self-consciousness; but it suffers nevertheless, for it adapts its actions to a determinate end. All this appears to us contradictory both to the ordinary meaning of words and to facts. Firstly, we cannot imagine any creature suffering without being conscious of pain; secondly, adaptation in the animal implies reasoning; thirdly, we know certainly that movements usually connected with highly pleasurable or painful feelings have occurred in paraplegic patients, without any suffering or feeling whatever on the part of the individual. We allude to examples in which sexual intercourse has been completed by persons of this class, and also to retraction of the legs on irritation of the insensible skin. But, in fact, Dr. W. Griffin grants the principle that determinate and adapted movements may take place without consciousness. He observes, "that the contraction of the iris on the admission of light, of the lids in winking, and of the uterus after death, in those cases in which labour is said to have been completed in the coffin, are sufficient to prove that sensation is not, in all cases, essential to the accomplishment of the action which usually succeeds it." This admission puts an end to the argument.

The question really with Dr. Griffin resolves itself into this, What *amount* of determinate and adapted movements may take place without sensation? Dr. Hall thinks more, Dr. Griffin less; but others go farther than either, as Dr. Griffin will have learnt already. New views as to the nature of the phenomena under discussion, and indeed, of mental phenomena generally, are gradually coming into notice. It is at all events certain that the adaptation spoken of is not done *by* the animal as a mental act, but are effected *in* the animal as the result of an inner mechanism adapted to external impressions. The closure of the eyelids, the contraction of the iris, &c. are examples.

PROBLEM X. "*What are the therapeutic effects of opium?*" The results of an opiate are sometimes sinking, and faintness without stupor, and occur many hours after exhibition. These symptoms

"May be removed or alleviated by renewing the opiate in a smaller dose, or, if other considerations render this unadvisable, by stimulants frequently repeated, and eventually by sleep. It is often of great importance in medical practice, and especially so in all dangerous cases where opiates may have been largely administered, that we should remember a period of exhaustion and sinking is likely to come on from twelve to twenty-four hours after the opiates have been discontinued. It almost invariably occurs where the opiates have previously been long continued, or taken in frequently repeated doses, or where there is a great constitutional susceptibility to their action. .... I advert to the subject chiefly on account of the danger or difficulty which often arises, when the physician either forgets the quantity of opium he has been giving, or is not sufficiently alive to the probable effects of its sudden withdrawal. Finding his patient sink, his countenance pallid and depressed, and his whole appearance betraying, sometimes, signs of excessive debility, he attributes the change to some unfavorable turn in the course of the disease, becomes alarmed for his safety, and, perhaps, resorts to medical measures either unnecessary or mischievous. This is particularly apt to occur in the cases of young children or infants, when the violence of the inflammatory symptoms has gone by, and it has been found necessary to administer opiates. About the time the influence of the opiate is wearing out, if it has been a large one, the eyes look sunk, the eyelids lie half open, disclosing a small portion of the white cornea, the face is deadly pale, the skin clammy, and the whole appearance of the child suggests an apprehension that it is dying. Yet if a little nourishment or some slight stimulant be given, or even if a little time be allowed to elapse, the heart will recover its tone, the little patient will revive, and may, perhaps, finally appear to be even in an improved condition." (pp. 189-90.)

Dr. Griffin has found that an overdose of opium will produce the same effect as an underdose; that is to say, a drowsy restlessness; but sound sleep would follow during the next night. He has found also, that "in administering opium, or indeed any narcotic whatsoever, for the relief of pain purely nervous, or connected with some condition of mere irritation and periodical in its occurrence, it will be found that a third part of the dose which may be found necessary to give relief in the paroxysm, will prevent it altogether if given in the interval, a little before its accession is expected. That, in fact, a moderate dose given in the interval will sometimes prevent the accession of the fit, when no quantity however great can control it, after it has once set in." (p. 193.)

We have already noticed Dr. W. Griffin's recommendation of opium as an antiphlogistic remedy. It is useful in inflammation of the mucous as well as the serous membranes; but in all cases full doses are the best. We would however put in a caveat: let the practitioner beware how he gives full doses to delicate invalids.

"The mischiefs arising from the administration of opium in inflammation may always be fairly attributed to the practice of under-dosing. In inflammations of serous membranes, which would be rapidly arrested by large doses, small ones incite and tend to increase the mischief,—in those of mucous membranes, small opiates do still more injury apparently, because while they are inefficient to diminish or arrest the inflammation they check or arrest the diarrhœa, which, however exhausting, is a relief so long as the inflammatory action continues unabated. A large opiate suppresses the inflammatory action and the diarrhœa at the same time; and, as a general principle, I think it will be found that the efficacy of its action will be in proportion to the degree of debility present. This would obviously suggest the propriety of abstracting blood in those cases where, from the violence of the arterial action, the antiphlogistic power of opium may possibly be resisted, or where on exhibiting it no relief has been attained. For my own part, in the treatment of most intestinal inflammations, whether of serous or mucous surfaces, I would confide more in the influence of blood-letting, large opiates, and warm poultices to the abdomen, than in all the remedies included in our materia medica." (p. 195-6 )

In a deplorable case of dysentery, Dr. Griffin, following a suggestion of Dr. Gregory's, gave three grains of opium with five of ipecacuanha at a dose, and directed it to be repeated every hour until some relief was experienced, and then continued every second hour until all pain and discharge ceased. "The effect was magical! Before he had the third dose taken my patient was asleep, and enjoyed more perfect and enduring relief than he had experienced for weeks."

"Of all the wonderful influences however exerted by opium, that by which it sustains the powers of life when sinking from hemorrhage, and arrests the flow of blood, is the most extraordinary. When after severe uterine hemorrhage the countenance is sunk, the eye hollow and glassy, the lips blanched, the skin cold, and the whole person corpse-like, when the pulse is almost gone at the wrist, when the beat even of the heart is scarcely perceptible, and stimulants, even brandy or rectified spirits, are either vomited or uninfluential, there remains yet one remedy capable of restoring the patient to life, and that is opium. I believe its power of saving life in these circumstances depends principally on its specific property of producing congestion in the brain. That amount of congestion by which it occasions apoplexy when given in large doses to persons in health, seems only sufficient to sustain the natural and necessary tension of the cerebral vessels in those who are dying of hemorrhage. Persons die in cases of hemorrhage, not so much from mere debility of the heart's action, as from the loss of nervous power in the brain consequent to it, and hence a fainting from which they are never awakened. The opium in such cases not only stimulates the heart's action, but restores a sufficient degree of tension in the vessels of the brain to prevent faintness, and by the judicious repetition of the remedy, life is preserved on the very borders of death. There are no instances in which opium can be given so freely or so fearlessly as in these. When the danger is imminent, five grains may be given at the first dose, and two or three every hour or half hour afterwards, until the pulse becomes distinct, the breathing easier, and the tossing or flinging about in the bed is allayed. It is hardly necessary to observe, that in such cases, in conjunction with the use of opium, the administration of warm wine and brandy, (however inefficient alone,) and the application of heat to the extremities, are highly useful, if not absolutely essential." (pp. 201-2.)

This is a very valuable paper, and fraught with truths of the greatest practical importance.

Problem XI. "*What principles should regulate the treatment of hemoptysis?*" This is a very useful paper, inasmuch as it is opposed to that system of routinism which subjects all cases of hemoptysis to an antiphlogistic treatment, with strict abstinence. Dr. W. Griffin relates some instances in which a contrary method was eminently successful. We could add corroborate facts from our own experience; and we are strongly convinced that a thorough revisal of the pathology of this affection is absolutely necessary to a discriminating and safe treatment. Dr. Griffin thinks that the starving method increases the tendency to hemorrhage, by rendering the vascular system more lax and irritable. The hypothesis is plausible; the fact that starvation increases the hemorrhagic tendency is certain. Another bad result of protracted low regimen and frequent depletion is referred to by Dr. Griffin,—the danger of inducing phthisis; and he thinks the need of such treatment is the less when an emetic of ipecacuanha, and the continued administration of it every hour, will subdue a frightful hemoptysis.

Problem XII. "*How should acute rheumatism be treated,* so as to effect a cure in the shortest space of time, with the least pain or other symptoms likely to protract or interrupt the cure, with the least probability of inflammation of a vital organ, and with the least after ill effects, whether debility, salivation, or chronic disease, and with the least liability to relapse?" Large and repeated bleedings, and the sweating and stimulant plans are going fast out of repute; colchicum is less certain, more painful, and sometimes attended with more serious consequences than other remedial agents.

"Emetic tartar sometimes acts like a charm," Dr. Griffin observes, "but when the practitioner tests the remedy more extensively, he finds, not only it is often attended with inconvenience, and if injudiciously pushed, with hazard, but that its failures are considerable in point of number. With Lepelletier they amounted to half those in which he employed it, and the success was little better even when accompanied by bleedings. In the Limerick Infirmary I believe the failures were more than two-thirds."

Finally, Dr. Corrigan's and Dr. Hope's plans are pronounced the best. The former administers one or two grains of opium every second or third hour. The medicine should be always increased in dose, both as to frequency and quantity, until the patient feels decided relief; and should be then kept up at that dose until the complaint is steadily declining. The first indication that tells the practitioner he has reached the proper dose is the statement of the patient, who in reply to an inquiry, as to how he has past the night, probably says that he has not slept, but that he is free from pain and feels comfortable. This effect having been attained, the opium may then be continued in repetitions of the same dose as to frequency and quantity. Dr. Hope gave from five to ten grains of calomel every night with from a half to two grains of opium, and in the morning a smart pugative. Three times a day a saline draught is also given, with ten to twenty minims of colchicum wine and five grains of Dover's powder.

The last essay in the volume before us is entitled, "*Observations on the application of mathematics to the science of medicine,*" and is written by the brothers conjointly. The object of the paper is to recommend the numerical method. Our views on this question have been already very fully stated.

We have now to sum up, and give our critical opinion on the essays before us. We judge then, firstly, that the writers are well-informed and sensible men, and careful, industrious, and observant practitioners; secondly, that they are better practitioners than physiologists; and thirdly, that their pathological theories are (consequently) marked more by ingenuity than by depth or comprehensiveness. To the practising physician and surgeon, however, the book will be found very useful, and will amply repay a careful perusal: we therefore strongly recommend it to all our readers.

The volume is full of typographical defects—partly, no doubt, the consequence of its provincial origin; and it contains some odd expressions, as for instance, "and directions were given if he *got any weakness* during the night to give him wine and water." We are sure that not long ago the very respectable authors must have regretted, as much as we did, to read a long and prominent notice of their book in a London morning paper. This must have been the work of some over-zealous and ill-judging friend, who did not consider that the legitimate road to professional reputation is through the *professional* public alone. A writer must be judged by his peers, if he would have a true judgment, and not appeal to an inadequate tribunal. The merits of this publication indeed rendered such an appeal quite unnecessary; its useful and practical tendency will secure it a favorable notice by the profession.

---

